# High genetic polymorphism of relapsing *P. vivax* isolates in northwest Colombia

**DOI:** 10.1016/j.actatropica.2011.03.012

**Published:** 2011-07

**Authors:** Eliana Restrepo, Mallika Imwong, Winston Rojas, Jaime Carmona-Fonseca, Amanda Maestre

**Affiliations:** a Grupo Salud y Comunidad, Facultad de Medicina, Universidad de Antioquia (Medellín, Colombia), Medellín, Colombia; b Department of Clinical Tropical Medicine, Faculty of Tropical Medicine, Mahidol University, Bangkok, Thailand; c Department of Molecular Tropical Medicine and Genetics, Faculty of Tropical Medicine, Mahidol University, Bangkok, Thailand; d Grupo Genetica Molecular, Instituto de Biologia, Universidad de Antioquia, Medellín, Colombia

**Keywords:** *Plasmodium vivax*, Malaria, Microsatellites, Polymorphism, Recurrence, Relapse, Colombia

## Abstract

Genetic diversity of *Plasmodium* populations has been more extensively documented in Colombia for *Plasmodium falciparum* than for *Plasmodium vivax.* Recently, highly variable microsatellite markers have been described and used in population-level studies of genetic variation of *P. vivax* throughout the world. We applied this approach to understand the genetic structure of *P. vivax* populations and to identify recurrence-associated haplotypes. In this, three microsatellite markers of *P. vivax* were amplified and the combined size of the fragments was used to establish genotypes. Patients from an ongoing treatment efficacy trial who were kept either in endemic or non-endemic regions in the northwest of Colombia were included in the study. In total 58 paired clinical isolates, were amplified. A total of 54 haplotypes were observed among the two regions. Some haplotypes were exclusive to the endemic region where the highest degree of polymorphism was detected. In addition, we confirmed the different genotypes of recurrent-relapsing and primary infection isolates suggesting the activation of heterologous hypnozoite populations. We conclude that analysis of the three microsatellites is a valuable tool to establish the genetic characteristics of *P. vivax* populations in Colombia.

## Graphical abstract

**Graphical abstract:**

**Figure:**
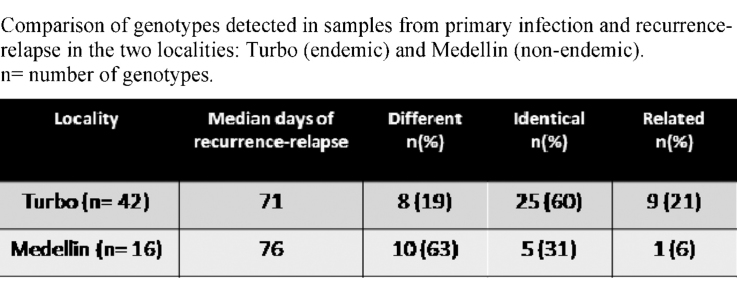

**Highlights:**

► Microsatellite markers were used to genotype *P. vivax* in of Colombia. ► A total 54 haplotypes in 58 paired (primary-recurrence/relapse) samples were found. ► Polymorphism in samples from endemic area was high. ► Relapsing and primary isolates had a different genetic conformation. ► The selected markers are useful to study *P. vivax* populations in the country.

## 1 Introduction

*Plasmodium vivax* is the most common species causing malaria in the Americas; in Colombia it is responsible for 65–75% of the cases of malaria in the country. The ability to distinguish between isolates and populations of *P. vivax* is a requirement for understanding the local and global epidemiology in order to implement practical and effective control strategies. The genetic diversity of *Plasmodium* populations has been better documented in the country for *falciparum* than for *vivax*, with some reports based on the analysis of *P. vivax msp1* and *msp3-alfa* genes (Cristiano et al., 2008; Imwong et al., 2007a; Maestre et al., 2004) or its proteins (Espinosa et al., 2003; Gutierrez et al., 2000; Martinez et al., 2005). In addition, neutral markers such as microsatellites (Imwong et al., 2007a) have been used to report differences in the degree of polymorphism in *P. vivax*. However, country-wide studies that apply similar protocols to evaluate genetic differences are lacking.

In clinical practice, *vivax* infections are treated with chloroquine (CQ) administration at the standard dose (10 mg/kg/day on day 1 and 7.5 mg/kg/day on days 2 and 3). However, chloroquine-resistant *P. vivax* has been reported worldwide (Baird et al., 1991; Garavelli and Corti, 1992; Garg et al., 1995; Kyaw et al., 1993; Marlar-Than et al., 1995; Murphy et al., 1993; Russell et al., 2003) and, more recently, high treatment failure rates of around 10% were described in the Amazon region (de Santana Filho et al., 2007). In Colombia, chloroquine-resistant *P. vivax* was detected in 2001 (Soto et al., 2001). However, the Ruebush et al. (2003) criteria to classify resistance to chloroquine are not fulfilled. In South America, these are met by two other studies: one reporting on a Guyanese traveler in 1996 (Phillips et al., 1996) and another reporting on two patients of the Peruvian Amazon in 2003 (Ruebush et al., 2003). In Colombia, the standard schemes CQ and CQ plus primaquine (PQ) have been effective in 95–100% of cases against blood forms of *P. vivax* (Carmona-Fonseca et al., 2006, 2008).

Recurrences are strongly associated to the administration of PQ and is well known that the interval between the primary infection and the relapse varies among the subtropical and tropical strains (Adak et al., 1998, 2001). Our studies confirmed a short period relapse pattern in this region of Colombia with 91% of relapses occurring between days 29 and 90 (Carmona-Fonseca and Maestre, 2009).

To prevent *P. vivax* relapses from persistent liver stages (hypnozoites), treatment with PQ is required at doses and schemes that vary between the different endemic regions. The standard dose of PQ currently recommended by the Colombian health authorities is 0.25 mg/kg/base for 14 days or 0.50 mg/kg/base for 7 days. During the past five years we have focused on the evaluation of the efficacy of alternative primaquine schemes against *P. vivax* malaria. We confirmed the improved performance of the administration of the standard total dose (STD) during 14 days (0.25 mg/kg/day/14 days) compared with the sub-dose over fewer days (3, 7, or 10 days) (Alvarez et al., 2006). In addition, the efficacy against recurrences was higher with the administration of the STD in 14 days than in 3, 5 or 7 days (Carmona-Fonseca and Maestre, 2009).

Compared with the more virulent parasite *Plasmodium falciparum*, knowledge about the genetic variability of *P. vivax* is limited, probably due to the emphasis on vaccine-oriented studies. Some studies have focused on the genotyping of *P. vivax* aimed not only at developing vaccine candidates against but also at unveiling specific genetic characteristics, and their association with pathogenesis, within regional contexts (Imwong et al., 2007a; Hernandez-Martinez et al., 2011). From these reports, it has become clear that evaluation of treatment response of *P. vivax* in areas of malaria transmission requires parasite genotyping at the local or regional level to distinguish homologous parasites from heterologous parasites in recurrent infections. Imwong et al. (2007b) have reported on the difficulties with interpreting molecular analyses of paired samples from primary and relapsing infections due to the presence of heterologous hypnozoite populations in patients with *vivax* malaria.

Microsatellites are the current markers of choice for large-scale population genetic studies and highly variable microsatellite markers have been recently described and used in population-level studies of genetic variation in this species (Carlton et al., 2008; Ferreira et al., 2007; Gomez et al., 2003; Imwong et al., 2006, 2007a,b; Karunaweera et al., 2007, 2008). In order to understand the molecular characteristics of *P. vivax* parasites from the northwest region of Colombia and to identify relationships between primary infection and recurrence-relapse isolates, we selected paired samples, from individuals who remained within the malaria endemic area throughout the follow up period, and from subjects who, after acquiring the infection in the endemic area, traveled to a non-endemic area where they remained after treatment and during the follow up period. Using microsatellite analysis, we identified the distinct parasite populations within these groups of patients.

## 2 Materials and methods

### 2.1 Study area

The study was carried out in the city of Medellin, which is non-endemic for malaria, and the malaria endemic municipality Turbo (8°5′42″N, 76°44′123″W). The region is mainly inhabited by people of African origin although mixing with indigenous and Spanish descendants can be observed (Carmona-Fonseca, 2003, 2004). *P. vivax* was responsible for 88% of malaria cases in Turbo during 2006–2007. In this municipality, malaria transmission is perennial and unstable (Carmona-Fonseca, 2003, 2004) with mean annual parasite indexes (malaria cases/1000 exposed individuals in one year) during 2006–2007 of 46 and 61, respectively (DSSA, 2007, 2008). Medellin (6°13′55″N, 75°34′05″O) is the second largest urban district of the country, with average temperatures of 20 °C, 1.538 m altitude and no malaria transmission.

### 2.2 Sample collection

In the endemic area, we conducted a randomized, non-masked, controlled clinical study on *P. vivax* infected patients. Volunteers were part of a larger study on the efficacy of different standard doses of CQ and different doses of PQ for prevention of recurrences in *P. vivax* infected patients (Alvarez et al., 2006; Carmona-Fonseca et al., 2009). Patients were recruited and randomized into one of three experimental groups with different treatment regimes. All subjects received the standard CQ regimen (10 mg/kg on day 1 and 7.5 mg/kg on days 2 and 3), and then received different PQ schemes. All treatments were supervised. Antimalarials were supplied by the regional health authorities and were administered once daily with 150–200 mL juice and a pastry. Samples were selected using the Lwanga and Lameshow methods (Lwanga and Lameshow, 1991) to test the differences in proportion between two independent populations (standard regimen of 210 mg total dose during 14 days vs. other). The initial sample size for each treatment was 49, and this was raised to 70 in order to compensate for dropouts during the lengthy follow up.

Inclusion criteria to participate in the recurrences study were unique *P. vivax* infection based in microscopy, attendance to controls, adequate treatment response, absence of recrudesce within the first 28 days of following up, and voluntary consent. Exclusion criteria included consent withdrawal or diagnosis of *P. falciparum* infection during the period of following.

In Medellin, the non-endemic region, samples were obtained from volunteers recruited at the malaria clinic of Universidad de Antioquia in a sequential order and a sample size was selected by convenience to obtain at least 15 subjects. All patients recruited in this locality received the standard CQ and PQ regimen (CQ 10 mg/kg on day 1 and 7.5 mg/kg on days 2 and 3 and PQ 0.25 mg/kg/day during 14 days). Patients recruited at this site acquired the infection at any of the major endemic areas of the country (Northwest and Pacific Coast) and attended the malaria clinic in Medellin to receive treatment.

Peripheral blood samples from patients attending the local malaria clinics with acute symptomatic *P. vivax* malaria and a positive thick smear between September 2003 and September 2004 were included. Single *P. vivax* infections were confirmed by diagnostic PCR using methods described elsewhere (Snounou and Singh, 2002). No distinction was carried out between relapse and re-infection. Therefore, both relapse and re-infection were included in the definition of recurrence.

Treatment response to CQ and PQ was assessed according to WHO guidelines (2006). For this, subjects were followed during the first 28 days, thereafter the frequency of recurrences was evaluated during 120 days after treatment. Any *P. vivax* parasitaemia after day 28 in patients with adequate treatment response was defined as recurrence.

The study protocol was reviewed and approved by the Ethics Committee of the Faculty of Medicine, Universidad de Antioquia (Medellín, Colombia). Each participant gave fully informed consent.

### 2.3 Preparation of parasite genomic DNA

Whole blood was obtained from individuals by venipuncture of the arm using Vacutainer^®^ tubes containing EDTA. One milliliter of blood was obtained from all patients on day 0 and on the day of the recurrence as evidenced by microscopy. *P. vivax* genomic DNA was purified using phenol/chloroform and stored at −20° until use.

### 2.4 Parasite genotyping and microsatellite PCR

For the evaluation of the circulating *P. vivax* haplotypes, defined by a combination of marker sizes of three microsatellite loci, genomic DNAs were amplified using a semi-nested PCR approach. Specific primers corresponding to the *P. vivax* repetitive sequences identified at TIGR as 14.297, 1.501 and 3.502, and located in chromosomes 14, 1 and 3, respectively, were used (Imwong et al., 2007b). Amplifications were carried out in a total volume of 20 μL, containing 2 μL DNA (1 μL of the product for the second PCR), 10 mmol/L Tris–HCl (pH 8.3 at 25 °C), 50 mmol/L KCl, 125 μmol/L dNTPs, 0.4 U Taq DNA polymerase (Fermentas), 2.5 mmol/L MgCl_2_ and 250 nmol/L of each oligonucleotide primer. The cycling parameters for PCR were as follows: initial denaturation was 5 min at 95 °C, followed by 20–25 cycles of denaturation at 94 °C for 30 s, annealing during 30 s at 52 °C and extension for 30 s at 72 °C. PCR products were stored at 4 °C prior to analysis. Paired samples were analyzed side-by-side on the same polyacrylamide (13%) gel. Product sizes were assessed under UV light after electrophoresis followed by ethidium bromide staining.

### 2.5 Determination of allelic types and mixed allelic types

For each microsatellite, size polymorphisms were assessed using the Quantity One^®^ 1-D Analysis Software (Bio-Rad). Allelic types for individual and combined markers were assigned to each sample. A sample was classified as having a mixture of alleles when multiple bands were present for any of the markers.

### 2.6 Comparison of acute infection and recurrence genotypes

Paired parasite populations were classified using the following criteria in the analysis of the three microsatellites: (a) different, none of the alleles detected before treatment were observed in the recurrence. (b) Identical, the same microsatellites detected before treatment were present in the recurrence. (c) Related, at least one of the same alleles was detected in samples before treatment and in the recurrence (Imwong et al., 2007b).

### 2.7 Data analysis

The virtual heterozygosity estimate (*H*_*E*_) was used as a measure of overall genetic diversity. This was defined as *H*_*E*_ = [*n*/(*n* − 1)][1 − Σ*p*_*i*_^2^], where *n* is the number of isolates analyzed and *p*_*i*_ is the frequency of the *i*-th allele in the population. *H*_*E*_ gives the average probability that a pair of alleles randomly selected from the population being different. Evidence of linkage disequilibrium between alleles from different loci in parasite populations was analyzed with ARLEQUIN v3.1 (Excoffier et al., 2005) available at http://cmpg.unibe.ch/software/arlequin3.

To represent the relationships between multilocus microsatellite haplotypes, a simple phylogenetic approach was used in which a median joining tree was built with Network v2.0. software (Bandelt et al., 1999), and a heuristic algorithm of maximum parsimony was applied. The program assumed a simple evolution model in which propagation and diversification of one haplotype in the population are established to build a correlation matrix.

Genetic measures of differentiation between regions were calculated as *Fst*-Statistic from haplotype frequencies; the null distribution of pairwise *Fst* values under the hypothesis of no difference between the populations was obtained by permuting haplotypes between populations and the *p*-value of the test was the proportion of permutations leading to a *Fst* value larger or equal to the observed one (Excoffier et al., 2005).

## 3 Results

In the endemic region, a total 42 subjects were recruited for a total of 84 samples collected at primary infection and recurrence (42 paired samples). In Medellin, 16 subjects were included for a total of 32 samples from primary infection and relapses. The mean period of the relapse-recrudescence was 71 days (±27). No infections by *P. falciparum* were detected during the 120 days of following up.

**Fig. 1:**
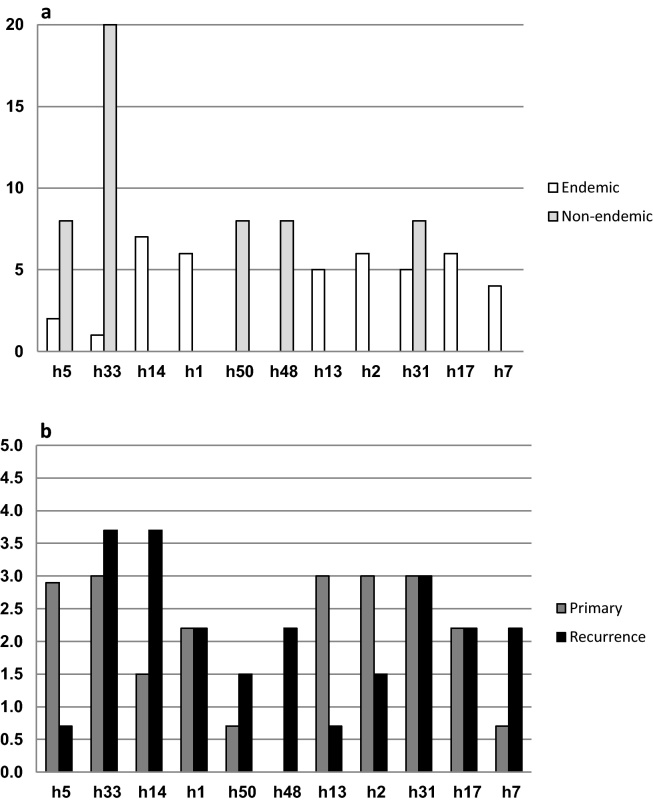
Comparison of proportions (percentage) of the haplotypes (h) with the highest frequencies according to locality of collection (panel a) and origin of the isolate (primary infection or recurrence-relapse, panel b).

The total number of haplotypes detected in all populations was 54, of which 37 were exclusive to the endemic region, 7 were exclusive to Medellin, and 10 haplotypes were shared in the two regions (see Supplementary Material). The haplotypes with the highest frequencies in each locality were different (Fig. 1), and only haplotype 31 was detected with a moderate frequency in samples from the two regions.

The mean prevalence presence of polyclonal infection, based on data obtained from at least one of the three loci was 13.7% (15.4% in Turbo and 9.3% in Medellin). The highest number of clones detected in one sample was 2 per loci, and in all cases this was evident only for one locus. In Turbo, polyclonality was confirmed in 8.3% for locus 1.501 and in 7.1% for locus 14.279. In Medellin, this was confirmed in a proportion of 3.1% for each locus.

Virtual heterozygosity (*H*_*E*_) in samples from Turbo was higher than in Medellin (Table 1). Presence of linkage disequilibrium for parasites from the endemic region was observed with the three microsatellites, and this was only evident for locus 1.501 in samples of Medellin (*p* < 0.05).

**Fig. 2:**
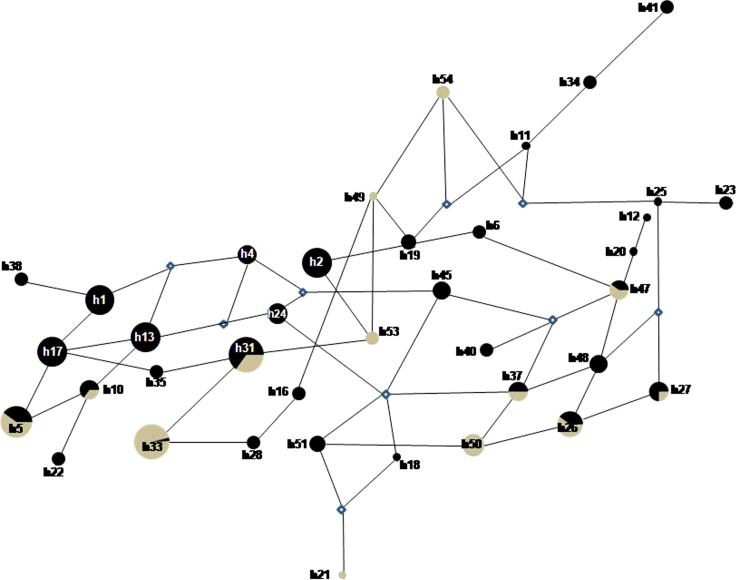
Relation between haplotypes (h) in Turbo (black) and Medellin (grey). Each circle represents a different haplotype and the size reflects the frequency. Related haplotypes are joined by a line and mutations (m).

Comparison of the genetic distances of samples from each locality according to *Fst* analysis, showed the low but significant differentiation between endemic and non-endemic region (*Fst* = 0.049 *p* < 0.0001). Haplotype clustering was only detected in samples from the endemic region (Fig. 2).

**Fig. 3:**
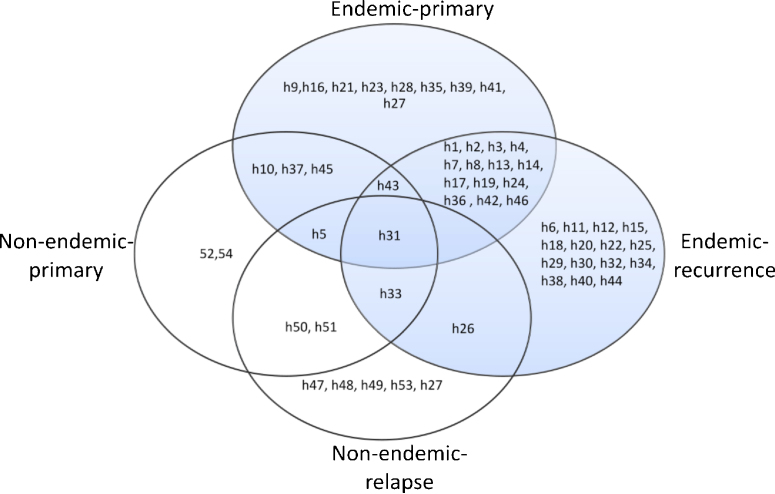
Distribution and relationship of the 54 haplotypes (h) among the two regions: endemic (grey) and non-endemic (white), according to the type of sample (primary infection and recurrence-relapse). Haplotype h27 was detected both in endemic primary and non-endemic relapse.

Global comparison of recurrent-relapsing and primary infection isolates from the two localities confirmed a high degree of polymorphism in isolates from recurrence-relapses (Fig. 1). In addition, greater polymorphism was detected in all samples from the endemic region when compared to all samples from Medellin. The highest diversity was detected in samples from recurrence in the endemic region. In the endemic region, seven different haplotypes showed frequencies around 5% and they represented over a third of all haplotypes. In the meantime, in the non endemic region, five different haplotypes were detected in around half of the samples. The reduced frequencies of individual haplotypes discriminated by region (endemic vs. non endemic) or PQ treatment received, impede a profound statistical analysis. The presence of exclusive haplotypes, either in primary infection or relapses, was detected in all regions (Fig. 3). Only haplotype 31 was detected in all regions, regardless of the primary infection or recurrence origin, and haplotypes 26, 31 and 33 were exclusive in recurrences. Comparison of primary infection and recurrence genotypes, based in the criteria described in Section 2, confirmed the presence of novel genotypes in 57% of the samples of Turbo and in 87% of samples of Medellin (Table 2).

## 4 Discussion

In this work, the genetic characteristics of *P. vivax* populations of northwest Colombia and a comparison of haplotypes of matched primary and recurrence-relapse isolates are described based on genetic analysis of three microsatellites. To our knowledge, this is the first study to explore the genotypes of relapsing and recurrent *vivax* isolates from this region of Colombia using microsatellites.

The frequencies of *vivax* malaria reported in the northwest of Colombia were among the highest in recent years. Between 2004 and 2009 in the locality of Turbo, Uraba region, a total of 24,713 malaria cases were reported, representing a mean annual *vivax* index of 32.4 per 1000 inhabitants (DSSA, 2010). During 2009, *vivax* malaria corresponded to 90% of the malaria cases of the region. Despite this high proportion of *P. vivax* in comparison with *P. falciparum*, few studies have addressed the genetic characteristics of the species. The most recent study identified polymorphisms in a rhoptry associated protein as part of vaccine candidate exploration. In this, only 11 samples were included from the northwest of the country (Garzon-Ospina et al., 2010). In 2008, Cristiano et al. published a study on the characteristics of *pvmsp3α*, a gene which has been reported by others as polymorphic and useful to characterize parasite populations. The authors reported 12 genetic patterns among 35 samples and recommended genotyping of microsatellites in future studies in order to overcome the bias introduced by the strong immune selection to which this gene can be subjected (Cristiano et al., 2008).

Different reports applied identical microsatellites as those used in the current study, and confirmed their efficacy to discriminate parasite populations in low endemic settings in Asia (Imwong et al., 2007b). Similarly, we were able to confirm the feasibility to discriminate *P. vivax* isolates of Colombia using these three microsatellites. Our results confirmed significant polymorphisms in a total 116 isolates obtained from primary infection and recurrence-relapse for a total of 54 different haplotypes detected. This correlates well when analysis of paired samples are made in other regions of the world and with non-paired samples studies from other regions in the Americas (Rezende et al., 2010). In addition, we confirmed the higher degree of genetic diversity in samples from subjects who remained in the endemic region compared to samples from subjects who move away from the endemic region, something not described in detail in other studies and that might be the result of a direct relationship between the degree of transmission and the number of haplotypes; alternatively, this might result of the low number of samples studied in Medellin.

Significant linkage disequilibrium was detected in Turbo after analysis of the three microsatellites, confirming the clonal structure of natural populations of *P. vivax*. This might be more likely explained by endogamy and/or natural selection, instead of physical relatedness. Our results are similar to those from Brazil (Ferreira et al., 2007) and Sri Lanka (Karunaweera et al., 2008) that report the presence of significant linkage disequilibrium and high haplotype diversity (*H*_*E*_ 0.71), confirming the clonal characteristics of the parasite populations in the presence of high genetic diversity. This can be explained by strand-slippage events during mitosis of the microsatellites studied. Endogamy can also explain the persistence of common circulating haplotypes, while polyclonal infections can be the result of abundant plus rare co-infecting clones.

In Turbo, the presence of exclusive haplotypes was confirmed and frequent. *Fst* analysis correlated well with the genetic relation analysis showing that, in the endemic region, haplotypes were closely related due to the reduced number of mutations among them, with new clones being generated from ancestral ones. *Fst* analysis in Turbo samples supports the theory of re-infection by the more common haplotypes, instead of relapse during the recurrence events. Meanwhile, the less frequent and disperse haplotypes might be associated with relapses.

In the non endemic area, exclusive haplotypes were also detected, but they were poorly related and, therefore, more dispersed. This might result from additional mutational events secondary to the diverse origin of the patients.

Analysis of shared haplotypes between the two regions confirmed that the less common ones in Turbo were common in Medellin. In addition, higher genetic distances were detected between these shared haplotypes, suggesting that some haplotypes of the non-endemic region originated from a common ancestral haplotype in Turbo.

Our results correlate with the observed low frequency of polyclonal infections in India but not in Thailand and Myanmar, in spite of the striking difference in endemicity between Turbo and India. A low frequency of polyclonal infection has also been reported by other researchers in Brazil (Ferreira et al., 2007) and Colombia (Imwong et al., 2007a), using different or additional genetic markers as reviewed by Havryliuk and Ferreira (2009). More recently, the application of more microsatellite markers revealed a high degree of diversity within and between Amazon parasite populations (Rezende et al., 2010). Altogether these results suggest a reduced frequency of polyclonal *P. vivax* infection in Colombia.

Transmission intensities in the Turbo are high and re-infection cannot be ruled out. However, in Medellin, re-infection was unlikely, so recurrences were certainly relapses. Imwong et al. (2007b) proposed that relapses resulted from a phenomenon of heterologous activation of hypnozoite populations. Similarly, our results, applying identical markers, suggest a different genetic structure of relapses-recurrences and primary isolates. This hypothesis has been controversial (Chen et al., 2007) but demonstration of similar results in samples from different countries might provide further support.

In the clinical practice, it has become more relevant to evaluate the efficacy of primaquine to prevent relapses. We were unable to demonstrate a particular genetic pattern in relapsing parasites. However, the data obtained can be applied to characterize *vivax* population dynamics within the different endemic regions of the country or the Americas region and/or can be of use to characterize different pathogenicity patterns (Dalla Martha et al., 2007). So far, the demonstration of selection for relapsing parasites after antimalarial treatment with primaquine to eradicate hyponozoites cannot be detected by application of these microsatellite markers and more sensitive genotype-specific detection methods might be required to resolve the question of whether initial infections contain more genotypes including relapsing associated ones.

Polymorphisms in *P. vivax* have been directly associated, by other authors, to time of sample collection with a large proportion of identical genotypes being collected on the same day (Imwong et al., 2007a). Exploration of temporal clustering of identical genotypes could not be performed in our study due to the lengthy period (up to 6 months) required for collection of paired recurrent-relapsing isolates. Recent studies in the Peruvian Amazon failed to confirm such phenomena in subjects followed for one year (Van den Eede et al., 2011). A different approach in which large numbers of samples can be obtained during short periods would contribute to further characterization of this phenomenon.

Other authors have reported on the limitation of PCR based assays to discriminate different clones within a mixture, with more abundant ones being preferentially amplified (Liu et al., 2008). This limitation in the technique might affect our results. The impact of such unequal amplification should be explored using a quantitative molecular approach such a qPCR.

Since the number of studies and genetic markers applied to Colombian samples has been very limited, more exhaustive analysis of clinical isolates from across the country should include application of genetic analysis either with more microsatellites or single nucleotide polymorphism analysis of markers which have been confirmed as very polymorphic in the Americas region (Orjuela-Sanchez et al., 2009, 2010).

## 5 Conclusions

We conclude that the three microsatellites are useful to establish the genetic characteristics of *P. vivax* populations in Colombia, resulting in a high degree of polymorphism in clinical isolates which was more clearly defined in samples from the endemic area. In addition, we confirmed the different genetic conformation of recurrent-relapsing and primary infection isolates. Based on these results we confirmed the presence of exclusive recurrence-related haplotypes in the endemic as well as the non-endemic region. We recommend to the research community interested in *P. vivax* to outline research priorities in order to better understand the genetic basis of the relapse phenomena.

## Figures and Tables

**Table 1:** Heterozygosity (*H*_*E*_), number of alleles per locus (*N*) and frequency (%) of the most abundant allele according to origin: Turbo (endemic) and Medellin (non-endemic). *n* = number of paired samples examined.

Locality	*H*_*E*_	Locus					
		1.501		3.502		14.297	
		*N*	%	*N*	%	*N*	%
Turbo (*n* = 42)	0.71	8	49	6	27	8	48
Medellin (*n* = 16)	0.60	4	50	4	59	3	50

**Table 2:** Comparison of genotypes detected in samples from primary infection and recurrence-relapse in the two localities: Turbo (endemic) and Medellin (non-endemic). *n* = number of genotypes.

Locality	Median days of recurrence-relapse	Different *n* (%)	Identical *n* (%)	Related *n* (%)
Turbo (*n* = 42)	71	8 (19)	25 (60)	9 (21)
Medellin (*n* = 16)	76	10 (63)	5 (31)	1 (6)
