# Supplementary material for: High genetic polymorphism of relapsing P. vivax isolates in northwest Colombia
Source: Acta Trop. 2011 Jul;119(1):23–9. doi: 10.1016/j.actatropica.2011.03.012 (PMC3485554; doi:10.1016/j.actatropica.2011.03.012)
Supplement: Supplementary file 1 [file mmc1.pdf]

Supplemental data.

Table. Detailed description of the haplotype denomination, origin, type of sample (recurrence-relapse) and dose of primaquine (PQ) administered according to clinical sample. Lack of amplification is denoted by (-).

Sample Code	Haplotype designation	Microsatellite			Total dose (mg) of primaquine administered
		1.501	3,502	14,297	
145	1	94	157	200	210
145r	1	94	157	200	210
146	1	94	157	200	150
146*	2	114	157	200	150
146rr	2	114	157	200	150
147	3	94	157	-	150
147r	3	94	157	-	150
150	2	114	157	200	150
150r	2	114	157	200	150
152	4	94	157	216	210
152r	3	94	157		210
153	5	94	133	200	105
153r	4	94	157	216	105
157	2	114	157	200	210
157r	6	114	157	216	210
158	7	94	-	200	150
158r	7	94	-	200	150
160	8	131	-	200	210
160*	9	70	-	200	210
160r	8	131	-	200	210
161	10	94	133	210	150
161r	11	131	157	210	150
161r	12	131	157	272	150
168	13	94	150	210	105
168r	14	94	-	210	105
168r*	15	94	-	272	105
172	16	114	133	210	105
172r	17	94	150	200	105
172r*	18	94	150	255	105
176	19	114	157	210	105
176r	19	114	157	210	105
176r*	20	114	157	272	105
177	10	94	133	210	150
177*	21	94	133	272	150
177r	22	94	113	210	150
178	23	200	150	220	105
178r	24	94	150	220	105
182	24	94	150	220	150
182r	24	94	150	220	150

Turbo	182r	25	131	150	220	150
	188	26	114	133	235	150
	188*	27	125	133	235	150
	188r	26	114	133	235	150
	189	28	100	133	210	150
	189r	29	100	-	210	150
	189r*	30	100	-	272	150
	191	14	94	-	210	105
	191r	14	94	-	210	105
	193	14	94	-	210	150
	193r	14	94	-	210	150
	198	17	94	150	200	105
	198r	1	94	157	200	105
	199	31	100	150	200	105
	199r	31	100	150	200	105
	201	5	94	133	200	150
	201r	33	100	133	200	150
	203	17	94	150	200	150
	203r	17	94	150	200	150
	223	13	94	150	210	210
	223r	34	200	157	200	210
	224	13	94	150	210	210
	224r	13	94	150	210	210
	230	2	114	157	200	210
	230*	1	94	157	200	210
	230r	14	94	-	210	210
	234	13	94	150	210	210
	234r	14	94	-	210	210
	237	35	70	150	200	210
	237r	17	94	150	200	210
	247	17	94	150	200	210
	247r	36	-	150	200	210
	252	31	100	150	200	210
	252r	31	100	150	200	210
	258	37	100	150	235	210
	258r	38	94	179	200	210
	259	39	100	-	235	210
	259r	40	100	179	235	210
	260	27	125	133	235	210
	260r	31	100	150	200	210
	261	41	200	133	200	210
	261r	32	-	133	200	210
	262	42	114	133	-	210
	262r	42	114	133	-	210
	265	43	100	-	200	210
	265r	43	100	-	200	210
	268	7	94	-	200	210
	268r	44	114	-	235	210
	269	45	94	157	235	210
	269r	1	94	157	200	210

	271	46	114	-	200	210
	271r	46	114	-	200	210
	272	43	100	-	200	210
	272r	44	114	-	235	210
	279	7	94	-	200	210
	279r	47	114	157	235	210
Medellin	1	31	100	150	200	210
	1R	26	114	133	235	210
	2	37	100	150	235	210
	2R	5	94	133	200	210
	3	33	100	133	200	210
	3R	48	114	150	235	210
	3R*	49	114	150	210	210
	4	10	94	133	210	210
	4R	48	114	150	235	210
	5	45	94	157	235	210
	5R	33	100	133	200	210
	6	50	100	133	235	210
	6R	50	100	133	235	210
	7	5	94	133	200	210
	7R	47	114	150	235	210
	7R*	48	114	157	235	210
	8	51	94	133	235	210
	8R	26	114	133	235	210
	9	43	100	-	200	210
	9R	27	125	133	235	210
	9R*	51	94	133	235	210
	10	52	94	-	235	210
	10R	53	114	150	200	210
	11	54	125	150	210	210
	11R	50	100	133	235	210
	12	5	94	133	200	210
	12R	47	114	157	235	210
	13	33	100	133	200	210
	13R	33	100	133	200	210
	14	31	100	150	200	210
	14R	31	100	150	200	210
	15	33	100	133	200	210
	15R	33	100	133	200	210
	16	33	100	133	200	210
	16R	33	100	133	200	210

*Second haplotype designation in case of polyclonal infection
